# Isolation of the Parasite *Enterocytospora artemiae* From Chinese Grass Shrimp (*Palaemonetes sinensis*)—First Report in Asia

**DOI:** 10.3389/fcimb.2020.580088

**Published:** 2020-12-07

**Authors:** Hongbo Jiang, Yuwen Chen, Jie Bao, Xiaodong Li, Chengcheng Feng, Yuenan Xing, Qijun Chen

**Affiliations:** Key Laboratory of Livestock Infectious Diseases in Northeast China, Ministry of Education, Key Laboratory of Zoonosis, Shenyang Agricultural University, Shenyang, China

**Keywords:** microsporidia, Palaemonetes sinensis, Enterocytospora artemiae, hepatopancreas, Enterocytospora-clade

## Abstract

Chinese grass shrimp (*Palaemonetes sinensis*) is an economically important crustacean in Chinese aquaculture. Recently, we found that shrimp in Panjin city were infected with microsporidia, a group of fungi. The hepatopancreas of several infected shrimp showed white turbidity and pathological changes that negatively affected the health and appearance of the shrimp. Histopathology and transmission electron microscopy were used to examine the development of the parasite within its parasitophorous vacuole. Our results indicated that microsporidia developed asynchronously within the same parasitophorous vacuole. The spores were predominantly small, and rod or oval-shaped. The sizes of fresh spores were approximately 3.1 × 2.4 μm and fixed spores were 1.9 × 1.1 μm. The polar filament was isofilar with 5–6 coils and the thickness was 103.2 nm. Merogonial divisions occurred by binary fission and sporogonial division occurred by plasmotomy. The small subunit ribosomal DNA sequence (1295 bp) from the parasite was highly similar to the previously reported parasite *Enterocytospora artemiae* (99% nucleotide identity, JX915760). Using maximum likelihood to analyze the phylogenetic relationships, we found that this microsporidian should be grouped within Clade IV, an *Enterocytospora*-like clade, of the Microsporidia phylum. Based on this parasite’s life cycle characteristics, morphology, and small subunit ribosomal DNA sequence, the parasite described here is likely *E. artemiae*, which has previously only been described in Europe and North America. Thus, this is the first report of *E. artemiae* both in Asia and economically important shrimp.

## Introduction

Microsporidia are single-celled eukaryotic pathogens that parasitize specific host cells. They are ubiquitous in the environment and can infect hosts from almost all known animal taxa (Dowd et al., 1998; Lom and Nilsen, 2003; Zhu et al., 2011). At present, more than 1,500 species of microsporidia have been identified globally (Weiss and Becnel, 2014). With the discovery of new hosts and infections, an increasing number of microsporidia will be found and identified (Chemurot et al., 2017; Simakova et al., 2018). Microsporidia are dangerous opportunistic pathogenic-microorganisms that very easily infect immunocompromised patients. Individuals with HIV, organ transplant patients, and patients on immunosuppressant drugs are particularly vulnerable to encephalitis and diarrhea caused by Microsporidia spp. (Wolf and Cowen, 1937; Galván et al., 2011). In addition, microsporidia infect silkworms, bees, salmon, shrimp, and other economically important and farmed animals (Lom and Nilsen, 2003; Wang et al., 2006; Klee et al., 2007; Sokolova et al., 2015), and have caused significant economic losses to the agriculture and aquaculture industries (Stentiford et al., 2016). More than 63 genera have been reported infecting crustaceans (Bojko et al., 2020), of which at least 15, including *Agmasoma*, *Ameson*, *Apotaspora*, *Enterocytozoon*, *Inodosporus*, *Myospora*, *Ovipleistophora*, *Paradoxium*, *Perezia*, *Pleistophora*, *Potaspora*, *Thelohania*, *Triwangia*, *Tuzetia*, and *Vavraia* have been identified in shrimp (Wang et al., 2013; Stentiford et al., 2015; Ding et al., 2016; Sokolova and Overstreet, 2018; Stentiford et al., 2018). Microsporidia infections within economically important crustaceans can lead to slow growth, muscle turbidity, hepatopancreatic lesions, and loss of economic value—all of which can seriously endanger the crustacean aquaculture industry (Tourtip et al., 2009; Wang et al., 2017).

Five major clades (I, II, III, IV, and V) and three taxonomic classes (Marinosporidia, Terresporidia, and Aquasporidia) of the phylum Microsporidia were identified and correlations between the major microsporidian clades and host habitat were analyzed by Vossbrinck et al. (2014). There are various microsporidia in Clade IV, including common Enterocytospora-like species, Enterocytozoonidae, and Hepatosporidae (Stentiford et al., 2011; Bojko et al., 2017). In recent years, an increasing number of important aquatic microsporidia, such as *Enterocytozoon hepatopenaei*, *Enterospora canceri*, and *Hepatospora eriocheir*, and also an important zoonotic pathogen of humans, *E. bieneusi* have been classified in this clade. *E. bieneusi* is closely related to *E. hepatopenaei* that infects the hepatopancreatic epithelial cells of *Penaeus monodon* and *Litopenaeus vannamei* (Tourtip et al., 2009; Kesavan et al., 2017). This close relationship attracts attention to the research focused on Microsporidia in aquatic crustaceans, which may be a possible evolutionary origin of *E. bieneusi* (Stentiford et al., 2019).

This study describes a novel microsporidian infecting the hepatopancreas of Chinese grass shrimp, *P. sinensis*, belonging to the Order Decapoda, Family Macrobrachiidae, and primarily distributed in China, Myanmar, southern Siberia, and Sakhalin. Owing to commercial trade, it has also appeared in Japan and other countries in recent years (Imai and Oonuki, 2014). Chinese grass shrimp not only have excellent sensory attributes but also have significant ecological value in lakes, rivers, and reservoirs. Recently, owing to the gradual depletion of its natural habitats, the aquaculture farming of Chinese grass shrimp can be of considerable economic benefit. In fact, Chinese grass shrimp has become an economically important crustacean, cultured in rice fields in China (Bao et al., 2018a). We first observed the microsporidial infections in Chinese grass shrimp collected from paddy fields and studied the morphology and ultrastructure of this microsporidian species. We also analyzed the full small subunit ribosomal DNA (SSU rDNA) sequence and performed a phylogenetic analysis, comparing it with those of other microsporidia in the National Center for Biotechnology Information (NCBI) public database. Based on ultrastructural and molecular evidence, we propose that this microsporidium parasite is synonymous with *Enterocytospora artemiae*, which has previously only been described within *Artemia* around Europe and North America (Rode et al., 2013).

## Materials and Methods

### Experimental Methods

Roughly 800 Chinese grass shrimp used in the experiment were collected from the paddy field of the Research Center of Panjin Guanghe Crab Industry Co., Ltd. (Panjin City, Liaoning Province, China). The shrimp were placed in oxygenated water in a plastic bag and cooled with ice for transportation to the Shenyang Agricultural University, Shenyang, Liaoning Province, China. There, they were kept in a circular 400-L water tank; the water was continuously aerated to ensure adequate oxygenation and was maintained at a temperature of 22 ± 1°C and a pH of 7.8 ± 0.2. The shrimp, which weighed 0.24 ± 0.03 g, were fed an artificial compound feed twice daily.

### Light Microscopy Observation

The external appearance and samples of the hepatopancreas and muscle of diseased shrimp were observed under a light microscope (LM) (Olympus-BX53, Tokyo, Japan). After photographing, hepatopancreatic smears from both diseased and healthy specimens were prepared and tested by PCR detection and sequencing to confirm their infection or non-infection by Microsporidia. Microsporidia were isolated from the infected hepatopancreatic tissue using the Percoll density gradient centrifugation method (Aldamacano et al., 2018).

### Histology

The infected hepatopancreatic tissue was fixed in 10% neutral formalin for 48 h. The sample was dehydrated by the ethanol and acetone gradient method and transferred to xylene to be rendered transparent. After embedding in wax, it was sectioned at 5 μm. LM was used for observation, and the sections were preserved after staining with hematoxylin and eosin.

### Preparation of Spore Suspension

Eighty samples of infected hepatopancreatic tissue were dissected and placed in a centrifuge tube containing sterilized water. The homogenate was again homogenized with a high-throughput tissue grinder (SCIENTZ-48, Ningbo Scientz Biotechnology Co., Ltd., Ningbo City, Zhejiang Province, China). After homogenization, the tissue fluid was filtered into the centrifuge tube, successively through 70-μm and 40-μm cell filters, and centrifuged (Xiangyi TGL-16M, Changsha, China) at 157.08 rad/s at 4°C for 10 min to collect the precipitate. The precipitate was added to sterilized water and centrifuged at 4°C for 30 s at 52.36 rad/s. The above operation was repeated twice. Finally, the precipitate was suspended in 1-ml sterile water after centrifugation at 4°C for 5 min at 314.16 rad/s.

### Scanning Electron Microscopy

The hepatopancreatic samples from diseased shrimp were trimmed to 3–5 mm, then fixed in 2.5% glutaraldehyde at 4°C and dehydrated in anhydrous ethanol gradient (50%, 70%, 80%, and 90% once for 15 min each time, and three times at 100% for 10 min), which was replaced with tertiary butanol for drying (50%, 75%, 90%, and 100% once for 10 min each time). After freeze-drying, gold coating, and specimen mounting, the samples were examined using a scanning electron microscope (SEM) (Hitachi Regulus 8100, Tokyo, Japan).

The purified suspension was fixed overnight with 2.5% glutaraldehyde, rinsed three times with phosphoric acid buffer solution, centrifuged in a high-speed centrifuge for 5 min at 10,000 r/min (1,047.2 rad/s), then embedded in filter paper and cut into 3–5 mm pieces. The following steps from dehydration were the same as above.

### Transition Electron Microscopy

The hepatopancreatic samples were cut into 1 × 1 × 1 mm cubes, fixed overnight with 2.5% glutaraldehyde, and rinsed three times with phosphoric acid buffer solution, for 15 min each time. The samples were then fixed with 1% osmium acid for 2 h and rinsed three times with phosphoric acid buffer solution for 15 min each time. Subsequently, samples were dehydrated by gradient alcohol dehydration (30%, 50%, 70%, for 15 min each time), and then with acetone gradient (80%, 95%, for 15 min each time; 100% three times, for 10 min each time). Finally, the samples were embedded in polymer resin (EMbed-812, Electron Microscopy Sciences, Ft. Washington, PA, USA), polymerized in an incubator at 60°C, cut into ultrathin sections (70–90 nm), stained with uranyl acetate and lead citrate, and were observed with a transmission electron microscope (TEM) (HT7700, Hitachi, Tokyo, Japan).

The spore suspension was fixed overnight with 2.5% glutaraldehyde and then centrifuged in a high-speed centrifuge at 1,047.20 rad/s for 5 min and rinsed three times with phosphoric acid buffer solution. The pathogen was embedded in agar and cut into 1 × 1 × 1 mm cubes. The following steps from dehydration were the same as above.

### DNA Extraction and Polymerase Chain Reaction Amplification

The hepatopancreatic DNA and 200 μl of the purified spore suspension DNA were extracted with the Qiagen DNA extraction kit (Beijing, China), in accordance with the manufacturer’s instructions. The 18S rDNA universal primers V1f and 1492r of Microsporidia were used for polymerase chain reaction (PCR) amplification. F: 5’-CACCAGGTTGATTCCTGAC-3’, R: 5’- GGTTACCTTGTTACGACTT-3’. The amplification process was as follows: denaturation at 94°C for 3 min, 35 cycles of denaturation for 45 s at 94°C, annealing for 30 s at 45°C, extension for 90 s at 72°C followed by 5 min extension at 72°C The PCR reaction system was in **Table 1**.

**Table 1:** PCR reaction system.

Reagent name	Volume (μl)
Sample DNA	1
Mix	13
Upstream primer	0.5
Downstream primer	0.5
dd H2O	10

Electrophoresis through a 1.5% Agarose gel (120 V, 30 min) was used to separate and visualize a resulting 1295 bp amplicon. Amplicons were recovered by TIANgel Midi Purification Kit (DP209, TIANGEN Biotech Co. Ltd., Beijing, China). The target fragment was connected to the T-Vector pMD™20 (Takara, Japan), and then transformed into the DH5α of competent cells. The cells were uniformly coated on to solid agar medium with 50 µl/ml of ampicillin and incubated overnight. Positive clones were isolated and commercially sequenced (Sangon Biotech Co., Ltd., Shanghai, China).

### Phylogenetic Relationship and Genetic Distance Analysis

The sequencing results were compared by basic local alignment search tool on NCBI (https://blast.ncbi.nlm.nih.gov/), and 18S rDNA sequence data and other species with high sequence similarity were selected from GenBank to construct the phylogenetic tree. The SSU rRNA gene sequences of 88 microsporidia were aligned by the E-ins-I algorithm within MAFFT (version 7). This alignment was analyzed for the best fitting model using Mega 7 (Kumar et al., 2016) and resulted in the GTR + G + I model choice according to BIC. The final tree was developed using a Maximum Likelihood process with 1,000 bootstrap replicates of the sequence data and had a log likelihood of −9619.0406.

## Results

### Gross Pathology

**Figure 1:**
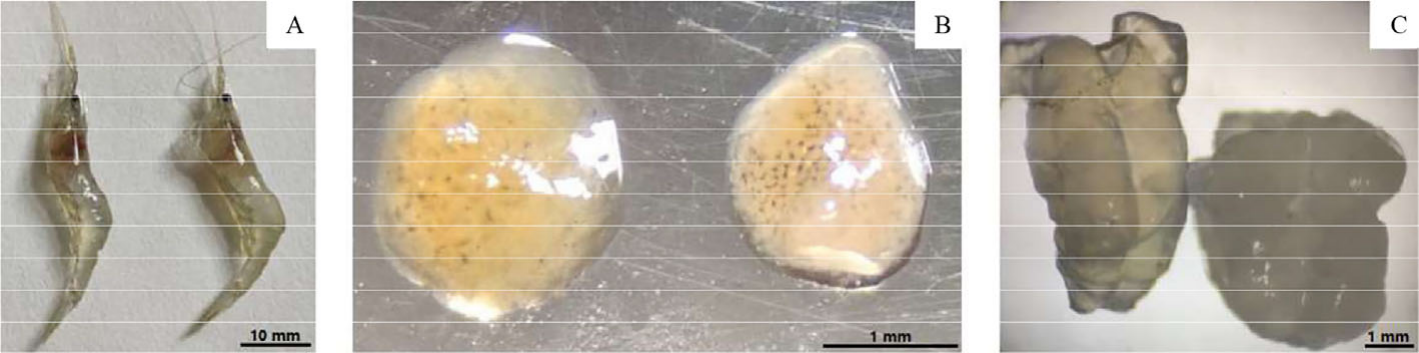
Comparison of uninfected (Left) and infected shrimp (Right). **(A)** External appearance; **(B)** Hepatopancreas; **(C)** Muscle.

Shrimp with moderate infection had no apparent symptoms, but specimens with severe infection (when spores were easily observed in a hepatopancreas smear) could be distinguished from healthy shrimp. The symptoms were evident from the external appearance of the shrimp, as shown in **Figure 1A**. The healthy shrimp body (left) was transparent, while the diseased shrimp body (right) was whitish with decreased transparency. The hepatopancreas of diseased shrimp was whiter than that of uninfected shrimp, and the black spots were darker and more numerous than those of healthy shrimp (**Figure 1B**). The muscle tissue of healthy shrimp was relatively transparent, while that of diseased shrimp was more turbid and opaque (**Figure 1C**). The color of the stomach, intestines, and heart of infected specimens showed no significant difference from those of the uninfected specimens.

### Light Microscope Observation

**Figure 2:**
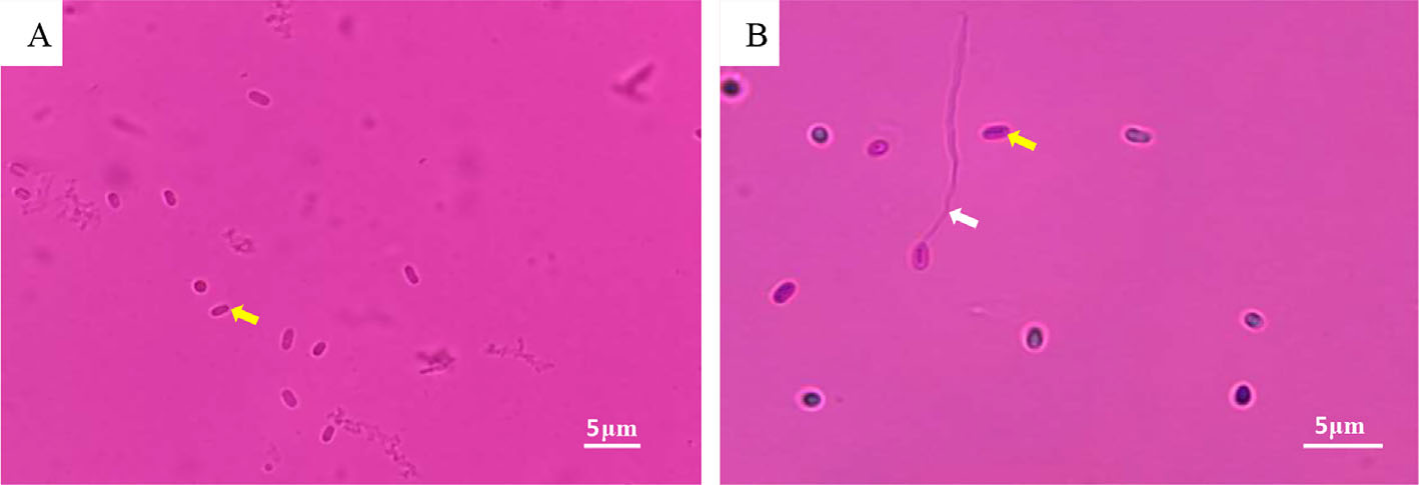
LM of Microsporidia. **(A)** Hepatopancreatic smear: spore (yellow arrow); **(B)** Purified Microsporidia: spore (yellow arrow); polar filament released by spore (white arrow).

**Figure 3:**
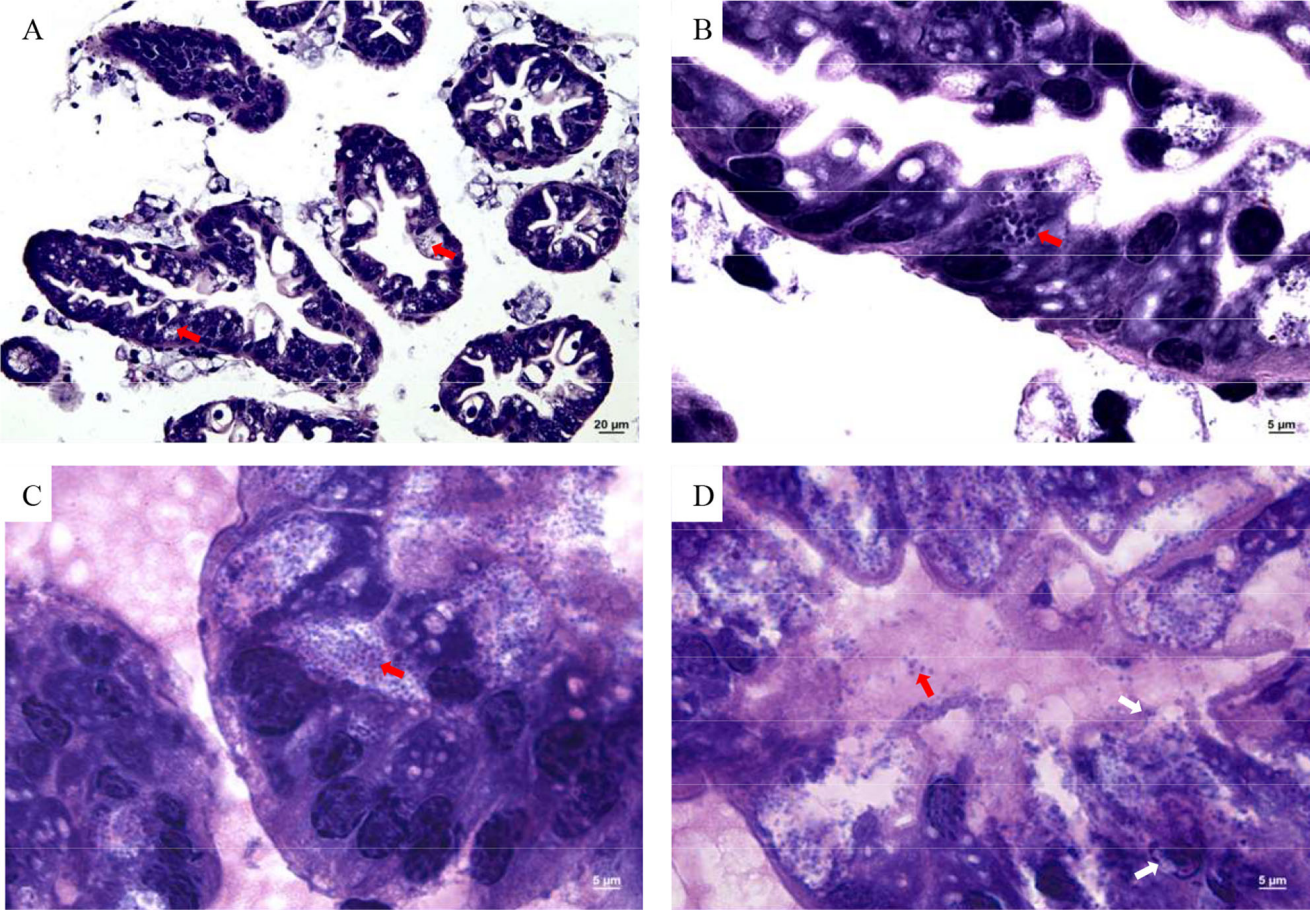
Microsporidia hepatopancreas tissue section. **(A)** 400×, (**B**–**D**) 1,000×. Red arrows indicate spores in cells and intercellular substance; white arrows indicate damaged cell membrane and nucleus.

Numerous Microsporidia, stained by phloxin B, were found in the hepatopancreatic smear, and the spores were purified by the Percoll density gradient (**Figures 2A, B**). The spores were predominantly small, and rod or oval-shaped. The size of fresh spores was approximately 3.1 × 2.4 μm. Polar filaments released from some spores were clearly visible (**Figure 2B**). The epithelial cells of the hepatic tubules were densely filled with spores (**Figures 3A, B**). A large number of spores were present, not only in the epithelial cells of the hepatic tubules, but also in the lumen after release from the cells (**Figures 3C, D**). The hepatopancreatic epithelial cells of diseased shrimp were swollen and some basic structures, such as the nucleus and cell membranes, had been destroyed (**Figure 3D**).

### Scanning Electron Microscope

**Figure 4:**
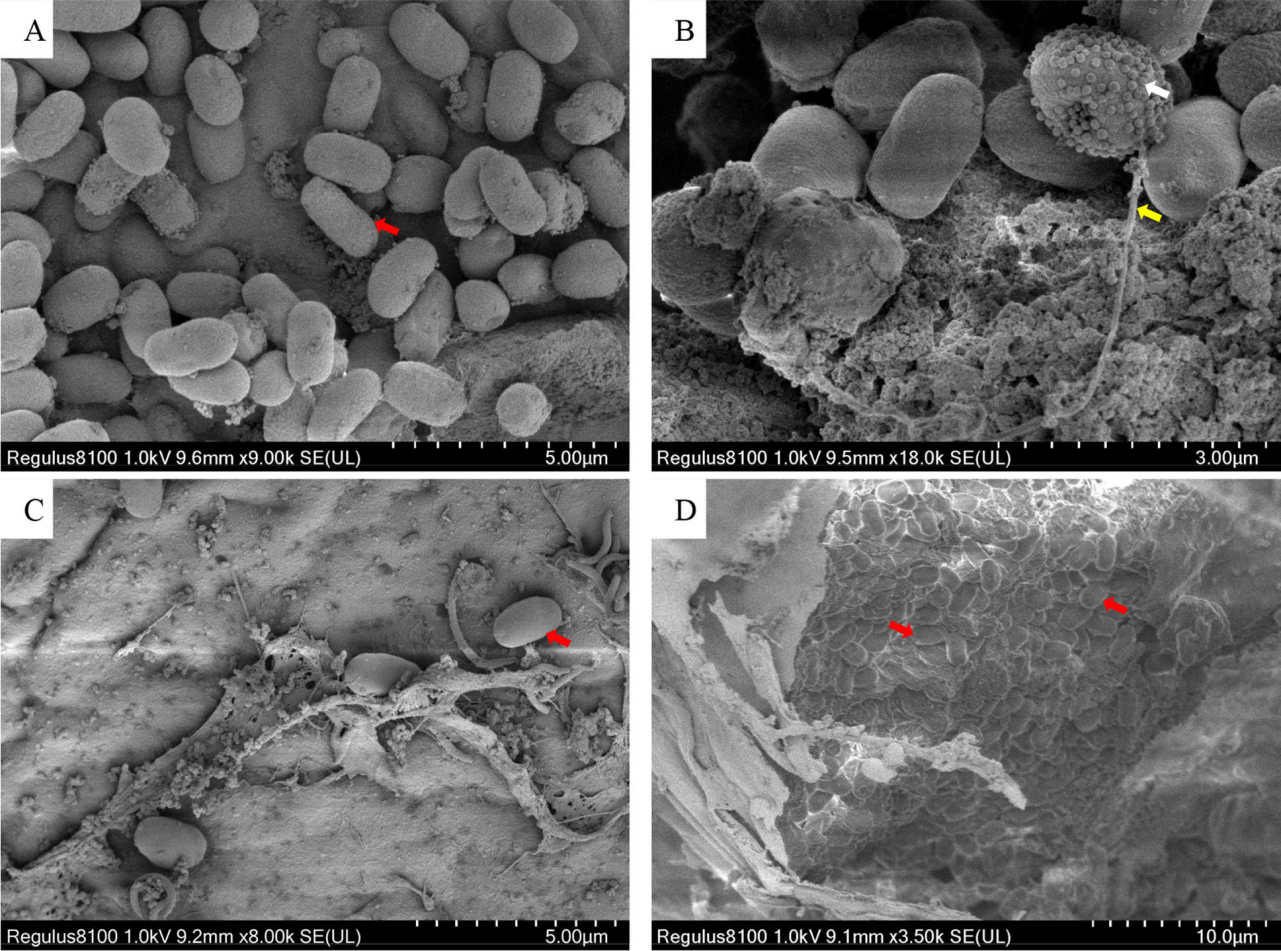
Scanning electron microscope photos of Microsporidia. **(A, B)** The SEM of purified spores. **(C, D)** The SEM of hepatopancreatic tissue. Red arrows indicate Microsporidia; the white arrow indicates spherical particles on the surface of a sporidium; the yellow arrow indicates the polar filament released from a spore.

As shown in **Figure 4**, the spores were predominantly small, and rod or oval-shaped (**Figures 4A, B**), with an average size of 1.9 × 1.1 μm. Spores were distributed in both intracellular and intercellular stroma (**Figures 4C, D**). Some spores released polar filaments and had spherical particles on their surface. Some cells had been entirely occupied by spores (**Figure 4D**).

### Transmission Electron Microscope

**Figure 5:**
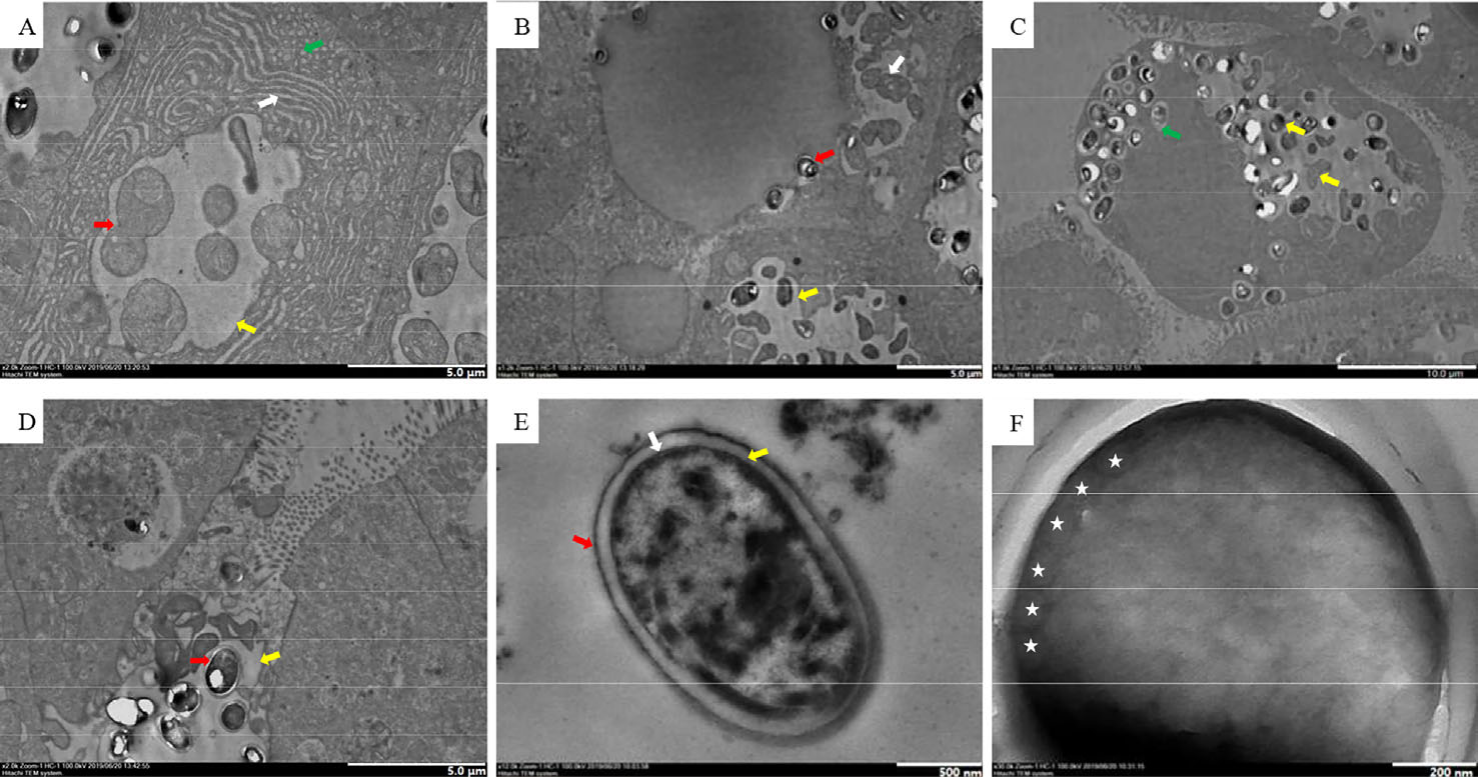
TEM of the Microsporidia life cycle. **(A)** Early stages of Microsporidia development, the red arrow indicates merogonial binary fission, the white arrow indicates the endoplasmic reticulum, the green arrow indicates a mitochondrion, and the yellow arrow indicates PV. **(B)** Sporogonial plasmotomy division (white arrow), the red arrow indicates a spore freed from the PV, the yellow arrow indicates PV. **(C)** The yellow arrows indicate different stages of spore development, and the green arrow indicates an isolated spore within the PV membrane. **(D)** Free PV in the hepatic lumen, the red arrow indicates a mature spore, the yellow arrow indicates PV. **(E)** Spore wall structure; the red arrow indicates the exospore, the yellow arrow indicates the endospore, and the white arrow indicates the plasmalemma. **(F)** Isofilar polar filament with 5−6 coils (asterisks).

The earliest stage observed was the merozoite, delimited by a single cytoplasmic membrane and included in a parasitophorous vacuole (PV) (**Figure 5A**). Generally, there was only one PV per cell. Merogonial divisions were by binary fission (**Figure 5A**). It was difficult to estimate the number of merogonial divisions. Sporonts and further developmental stages remained within the PV. Sporogonial division was by plasmotomy (**Figure 5B**), and mature spores remained in the same vacuole as the sporoblasts (**Figure 5C**). Some mature spores were enveloped in the PV membrane (**Figures 5B, C**), and PV were observed in the hepatic lumen (**Figure 5D**). Spores were subspherical and unikaryotic. The spore wall was divided into three layers (**Figure 5E**). The electron density of the exospore was high, and was approximately 14.8 nm thick. The endospore was an electron transparent layer that was 82.8-nm thick (thicker than the outer layer). The plasmalemma was the innermost part. There were 5–6 coils in the polar tubes (**Figure 5F**) and the angle between the polar tube and the long axis was 41.7°; the polar tubes were arranged in the same row, and thickness was 103.2 nm.

### Polymerase Chain Reaction Amplification and Sequencing Analysis

**Figure 6:**
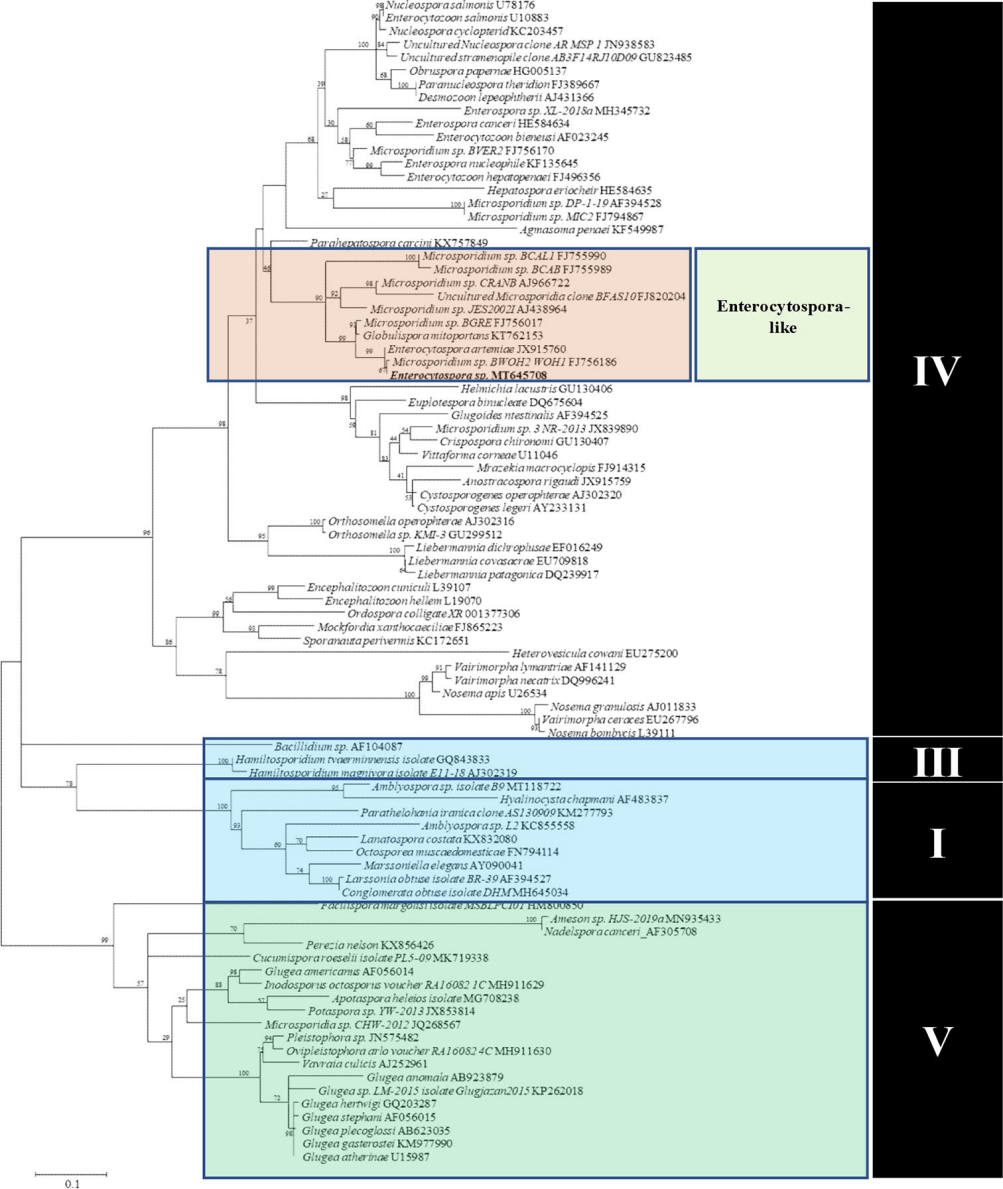
Maximum Likelihood analyses based on SSU rDNA sequences of Microsporidia and other microsporidian species. Scale bar corresponds to 0.1 substitutions per site. GenBank accession numbers follow the species names.

A single consensus DNA sequence (1,295 bp) from the Microsporidia parasite was obtained and was utilized to assess the phylogeny of the novel taxon. The BLASTn results obtained showed the highest similarity with the previously reported *E. artemiae* (99% nucleotide identity and 96% query cover, JX915760). Other similar species were *Globulispora mitoporans* (94% nucleotide identity and 96% query cover, KT762153), Microsporidium sp. PT11 (87% nucleotide identity and 88% query cover, KP966297), Microsporidium sp. BWOH2 WOH1 (99% nucleotide identity and 55% query cover, FJ756186), Microsporidium sp. I haplotype 1 (87% nucleotide identity and 82% query cover, KR871371), *Nucleospora salmonis* (83% nucleotide identity and 96% query cover, AF185996), and *Nucleospora cyclopteri* (83% nucleotide identity and 95% query cover, KC203457). The above results suggest that the new parasite belongs to Clade IV of the Microsporidia (Vossbrinck and Debrunner-Vossbrinck, 2005). Maximum likelihood analyses grouped this parasite within the branch of the *Enterocytospora*-like clade (Vavra et al., 2016; Bojko et al., 2017) (**Figure 6**).

## Discussion

The traditional biological classification standards of Microsporidia were mainly based on the life cycle, size, internal structure, spore morphology, and infectivity. At the molecular level, the SSU rDNA gene and its sequence are important marker DNA sequences for molecular classification research on Microsporidia (Dong et al., 2009). Therefore, this study on the molecular phylogeny of the microsporidian parasite infecting *P. sinensis* was primarily based upon a partial SSU rDNA gene. The top hits from the BLASTn search included *E. artemiae* isolated from *Artemia parthenogenetica* and *G. mitoportans* isolated from *Daphnia pulex*. Cluster analysis showed this parasite to belong to the *Enterocytospora*-like clade (Vavra et al., 2016; Bojko et al., 2017), and based on Vossbrinck and Debrunner-Vossbrinck (2005), can be classified as Clade IV of Microsporidia. Clade IV is represented by both the family Enterocytozoonidae and Hepatosporidae. Many Clade IV Microsporidia have caused significant damage to economic crustaceans, such as *Enterocytozoon hepatopenaei* to *L. vannamei*, and *Triwangia caridinae* to *Caridina formosae* (Tourtip et al., 2009; Wang et al., 2013). Although there was no mortality in Chinese grass shrimp infected with microsporidia, there were significant pathological changes in the hepatopancreas that had an adverse effect on their health and appearance.

From the perspective of the life cycle, all stages of this parasite occurred within the PV. This aspect is similar in *E. artemiae*, *H. eriocheir*, and *Inodosporus octospora* (Azevedo et al., 2000; Stentiford et al., 2011; Rode et al., 2013). The species *Thelohania montirivulorum* and *Tuzetia weidneri* develop in direct contact with the host cell cytoplasm during early stages and later become isolated from the host cell cytoplasm by the parasite-produced membranes at the sporogonic phase (Canning et al., 2002; Moodie et al., 2003). Species of the genera *E. hepatopenaei* and *Myospora metanephrops* develop in direct contact with the host cell cytoplasm (Tourtip et al., 2009; Stentiford et al., 2010). The development of this parasite is asynchronous with sporonts, sporoblasts, and mature spores found together within the same PV. Although different *H. eriocheir* PVs are not synchronized within the same cell, development within the same PV occurs synchronously (Stentiford et al., 2011). PV containing mature spores can be released intact into the hepatic lumen. The proliferative stage includes both cell growth and proliferation. Different genera follow different division patterns; in some genera, cells divide by binary fission (Cali et al., 1998). In other genera, multinucleate cells were produced by multiple nuclear divisions or by disintegration from a long strip of nuclei, without cytoplasmic division (Cali and Takvorian, 2003). In this study, merogonial divisions of this microsporidia occurred by binary fission, and sporogonial division occurred by plasmotomy. Based on its life cycle characteristics and SSU rDNA sequence analysis, the parasite described here is likely *E. artemiae*, which was first isolated from gut of species and named by Rode et al. (2013). *Enterocytospora artemiae* primarily infects the gut of *Artemia*, and has not been previously found within the hepatopancreas of Chinese grass shrimp. Morphologically, *Artemia* dwelling *E. artemiae* is slightly different from the microsporidian described in this study. Within *Artemia*, the average size is 1.2 × 0.9 μm, the thickness of the spore wall is 80 nm, and the number of polar tube coils is four (Rode et al., 2013). Whereas the average size of microsporidia in Chinese grass shrimp is 1.9 × 1.1 μm, the spore wall is 98 nm thick, and the number of polar tube coils is 5–6. This morphological discrepancy may be related to host or parasitic tissue difference. Microsporidians are highly plastic parasites and their morphological structures change greatly with host taxa (Kelling and Lipa, 1960). Even within the same host species, morphological differences occur due to different parasitic tissues. For example, *Ameson pulvis* within *Carcinus maenas* can produce two different types of spores, either needle-like spores when in the peripheral sarcoplasm of heart and skeletal muscle fibers or typical *Ameson*-like spores when in the skeletal muscles (Stentiford et al., 2013). Therefore, morphological differences we observed may be related to long-term parasitic adaptation to different hosts and tissues.

The previous known range of *E. artemiae* suggests a key infection source for Chinese grass shrimp. Chinese grass shrimp are species indigenous to China. Therefore, it is likely that this microsporidian is a native, rather than invasive, pathogen. Artemia, as important prey for fish and crustaceans, often transmit pathogens (Moore and Strom, 2003). The Chinese grass shrimp is mainly found in freshwater (i.e., rivers and lakes), but is able to tolerate waters with higher salinity (Bao et al., 2018b). However, this shrimp has not migrated between fresh water and salt water (Takakiyo et al., 2010). *Artemia*, on the other hand, thrive in water with high salinity levels. Therefore, the niche difference between these two hosts makes transmission between their natural habitats unlikely. Frozen *Artemia* are often used as artificial feed when culturing shrimp. We have tested several batches of commercially available frozen *Artemia*, but no *E. artemiae* was detected. Therefore, we speculate that the microsporidia in Chinese grass shrimp is not spread by *Artemia*. The infection source of *E. artemiae* in Chinese grass shrimp, and whether this parasite can be transmitted to other crustaceans, requires further study.

## Taxonomic Summary

### *Enterocytospora artemiae*

Type host. The freshwater Chinese grass shrimp *Palaemonetes sinensis* (Crustacean: Decapoda).

Transmission. Horizontal and vertical propagation (Data not published).

Site of infection. Hepatopancreas.

Interface. Parasitophorous vacuoles are generated from the initial merogony, and then, spores continue to develop inside the vesicles. Developmental stages are not in direct contact with the host cytoplasm.

Merogony. Merogonial divisions of *E. artemiae* occurred by binary fission.

Sporogony. Sporogonial division occurred by plasmotomy. The number of mature spores in PV was uncertain.

Spore. Mature fresh spores measure 3.1 × 2.4 μm. Fixed spores are 1.9 × 1.1 μm. Five to six polar filament coils of diameter approximately 103 nm. The spore wall consists of a 15 nm electron-dense exospore and 83-nm electron-lucent endospore.

Type location. (121.8503373800E, 40.9027472700N), Xinrongxian, Dawa District, Panjin City, Liaoning, China.

Molecular data. GenBank Accession No. MT 645708 for SSU rDNA.

## Conclusion

Chinese grass shrimp (*Palaemonetes sinensis*) cultured in Panjin city, China, were infected with microsporidia. Based on this parasite’s life cycle characteristics, morphology, and SSU rDNA sequence, it is likely that the parasite described here is *Enterocytospora artemiae*, previously only described within *Artemia* in Europe and North America. This is the first discovery of *E. artemiae* within both Asia and economically important aquacultural products.

## Data Availability Statement

The datasets presented in this study can be found in online repositories. The names of the repository/repositories and accession number(s) can be found at: https://www.ncbi.nlm.nih.gov/genbank/, MT645708.

## Ethics Statement

The animal study was reviewed and approved by Animal Experiments Ethics Committee of Shenyang Agricultural University.

## Author Contributions

HJ, YC, and QC were involved in designing of the research and wrote the manuscript. HJ, YC, JB, and XL performed the majority of the experiment, data processing, analysis, and interpretation. CF and YX assisted in sample collection and TEM observation. All authors contributed to the article and approved the submitted version.

## Funding

This work was supported by the Modern Agro-industry Technology Research System (CARS-48), Liaoning Province Key R&D Planning Guidance Plan Project (2019JH8/10200018), Liaoning province Department of Education fund item (LSNQN202002), Liaoning Science and Technology Mission Project (2020JH5/10400147), and Liaoning Province Key R&D Planning Project (2019JH/10200006).

## Conflict of Interest

The authors declare that the research was conducted in the absence of any commercial or financial relationships that could be construed as a potential conflict of interest.
